# A randomized, blinded, controlled USA field study to assess the use of fluralaner tablets in controlling canine flea infestations

**DOI:** 10.1186/1756-3305-7-375

**Published:** 2014-08-16

**Authors:** Cheyney Meadows, Frank Guerino, Fangshi Sun

**Affiliations:** Merck Animal Health, Summit, NJ USA

**Keywords:** Fluralaner, Fleas, Spinosad, Efficacy, Safety, Field study

## Abstract

**Background:**

The novel isoxazoline molecule fluralaner provides 12 weeks activity against fleas and 8 to 12 weeks against tick infestations according to label claims.

**Methods:**

This blinded, multi-center study in client-owned dogs evaluated the flea control provided by a single oral fluralaner treatment (25–56 mg/kg; Bravecto™, Merck Animal Health) compared to a control group administered three oral spinosad (30 – 60 mg/kg; Comfortis^®^, Elanco) treatments at 4-week intervals together with an amitraz collar (9%, Preventic^®^, Virbac). Households were randomized (3:1 ratio) to either fluralaner (224 dogs, 118 households) or control (70 dogs, 39 households). Within households, one primary dog with at least 10 live fleas at enrollment was randomly selected for whole body flea counts every 4 weeks through Week 12; all dogs were followed for safety until Week 12. Fluralaner dogs received two additional doses at Weeks 12 and 24 for further safety and palatability observations through Week 26.

**Results:**

Geometric mean flea count reductions from baseline for the fluralaner group at Weeks 4, 8, and 12 were 99.7%, 99.8%, and 99.8%, respectively; and 96.1%, 99.5%, and 99.6% for the spinosad controls. Percentages of flea-free primary dogs at Weeks 4, 8, and 12 were 91.1%, 95.4%, and 95.3% for the fluralaner group; and 44.7%, 88.2%, and 84.4% for the controls; the differences were significant at Weeks 4 (P < 0.0001) and 12 (P = 0.0370). Improvements in veterinarian assessed flea allergy dermatitis (FAD) were observed in both groups. Fluralaner tablets were accepted free choice in over 90% of doses. The most common adverse event was vomiting, occurring in 7.1% of the fluralaner group and 14.3% of the controls. No treatment related serious adverse events were reported.

**Conclusions:**

A single treatment of dogs with the palatable fluralaner flavored chewable tablet provides a safe and effective option for 12 weeks of flea control at least equivalent to that of 3 sequential treatments with spinosad tablets. Linked to the high level of flea control was a substantial alleviation of associated signs of FAD.

## Background

The last three decades have seen significant advances in the treatment and control of canine ectoparasites. Topically applied pesticides, such as imidacloprid and fipronil, were introduced in the 1990’s, and provided convenient month long flea control and, for fipronil, additional activity against a range of tick species [1]. The use of such topically applied products became a routine preventive health practice in veterinary medicine. These products, and others that were later introduced, either as single entities or as combinations of molecules, reduced or eliminated existing flea infestations and, for the month following treatment, killed most reinfesting fleas before egg-laying could begin. As a result, the need for adjunctive application of environmental chemicals was largely eliminated [2]. However, these topical ectoparasite control advances presented limitations, including the need for careful and sometimes challenging application by pet owners, potential reductions in effectiveness resulting from wash-off during swimming or bathing, and owner concerns regarding exposure in the household [3]. In 2007, an orally administered pulicide (spinosad, Comfortis, Elanco) was launched that provided an alternative means of killing fleas for one month following treatment [4]. While oral spinosad resolved many concerns linked to topical products, it lacked efficacy against ticks and maintained the need for owner compliance with monthly administration.

Fluralaner is a novel isoxazoline insecticide/acaricide that offers the most recent advance in the control of ectoparasites of dogs, with 12 weeks of flea control and 8 to 12 weeks of tick control after a single oral dose. This treatment was shown in a multi-centered field trial in Europe to be superior to three sequential doses of fipronil for flea control and also effective for tick control [5].

Bravecto (13.64% w/w flavored chewable tablet formulation of fluralaner, Merck Animal Health) is an oral fluralaner formulation that is the first product approved by the US Food and Drug Administration (NADA 141–426) to provide 12 weeks activity against fleas and 8 to 12 weeks activity against the 4 common tick genera in the United States (*Amblyomma*, *Ixodes*, *Dermacentor* and *Rhipicephalus*) at a minimum dose of 25 mg/kg. Following oral administration to dogs with or without food, fluralaner is rapidly absorbed, and provides 100% effectiveness against fleas and ticks (*Ixodes ricinus*) within 1 day after treatment [6, 7]. Blood levels are then sustained and provide flea and tick effectiveness >95% for up to 12 weeks [6, 7]. Safety has been demonstrated in puppies administered repeated doses of up to 280 mg/kg (five times the recommended rate) at 8-week intervals, beginning at 8 weeks of age, and in ivermectin-sensitive collies administered doses of 3 times the approved dose rate [8, 9].

The current field study was undertaken in the United States to confirm the effectiveness over 12 weeks (84 days) of a single owner-administered fluralaner flavored chewable tablet dose to treat and control flea infestations. These results were compared to that of a control group treated with spinosad and an amitraz collar. Evidence of flea allergy dermatitis (FAD) was not an enrollment criterion, but secondary objectives included an assessment of improvement from baseline in signs of FAD in a subset of dogs that presented with signs of FAD. Furthermore, safety and palatability of fluralaner tablets were assessed through 26 weeks (182 days) with dogs receiving an additional two treatments at 12-week intervals. Observational assessments of the tick control provided by each study regimen were also completed.

## Methods

This investigator-blinded, multicentric, positive-controlled study was undertaken to assess the flea control effectiveness of fluralaner tablets administered by owners to their flea-infested dogs. Comparisons were made with a control group receiving three sequential treatments of orally administered spinosad at 4-week intervals plus an amitraz collar. The study protocol complied with Good Clinical Practice (VICH GL9), the International Guiding Principles for Biomedical Research Involving Animals, and was reviewed and approved by an ethics committee. Written informed consent was obtained from each dog owner prior to the commencement of any screening activities. The study was conducted from August 2011 through June 2012 at 18 veterinary clinics located in nine different states – Alabama, Florida, Kansas, Louisiana, Maine, Missouri, North Carolina, Pennsylvania, and Texas. At each site, the investigator was the clinic veterinarian who was responsible for protocol supervision, oversight of owner communication, animal examination including flea allergy dermatitis (FAD) assessments, and supervision of flea counts. To maintain appropriate blinding, the flea treatment product assigned to each dog was dispensed by non-blinded trained clinic personnel who were instructed not to participate in collecting flea count data, physical examinations, or in the assessment of FAD. These personnel were also responsible for any additional measures required to ensure blinding, such as removing the amitraz collar on enrolled control dogs before they were examined by blinded personnel. Collars were replaced by owners or non-blinded personnel once blinded activities were completed.

### Enrollment and participation

Eligible households were permitted to have up to 5 dogs, all of which had to be at least 12 weeks of age, weigh at least 4.4 lb, and be in general good health. However, dogs with chronic medical conditions (e.g., endocrinopathies, cardiovascular conditions, seizures) were permitted to enroll if the condition was stabilized prior to enrollment in the study. There were no breed or gender restrictions, but households with pregnant or lactating dogs were not eligible for enrollment. Households in which dogs had exposure to non-confined pets (other than dogs) that could harbor fleas (e.g., cats) were not eligible. Similarly, households were excluded if they contained another dog which did not meet inclusion criteria.

Whole-body flea counts lasting at least 15 minutes per dog were performed on all dogs by a trained individual masked to treatment assignment. The criterion for household enrollment was that at least one dog in each household be infested with a minimum of 10 live fleas at the screening assessment.

The following signs of FAD were separately graded by a masked veterinarian (No Sign, Mild, Moderate, or Severe): erythema [10, 11], alopecia [11], papules [10, 11], scales [10], crusts [10] and excoriations [11]. When present (i.e., Mild, Moderate, or Severe), each sign was further assessed by the masked veterinarian concerning anatomic location and whether or not the sign was indicative of FAD. There was no protocol definition of FAD, and the veterinarian used their experience and knowledge to determine if signs were indicative of FAD. Evidence of FAD was not used as a criterion for enrollment or randomization.

Enrollment restrictions based on prior use of flea control medications and treatments were based on product label. Products labeled for monthly use had a minimum 30 day washout, products labeled for use every two weeks had a 14 day washout, and products labeled for weekly use had a seven day washout. Treatment that could affect assessment of signs of FAD (for example steroids, antihistamines, creams, ointments, baths, *etc*.) was permissible, but any FAD data collected after a dog was treated with such a product were excluded from summary and analysis of FAD data.

No concomitant treatments for flea and/or tick infestations were permitted during the study period. Grooming, bathing, swimming, and other water activities were permitted during the study, although participating dog owners were asked to temporarily remove amitraz collars prior to bathing. Grooming and bathing were not allowed within 72 hours before protocol-scheduled flea counting to avoid any impact on flea recovery.

To assess palatability of fluralaner tablets, owners were instructed to first offer the dose by itself, for instance by hand or in a bowl. The owner recorded if the dose was freely taken within 1 minute or within 1 – 5 minutes. If not, the owner could take alternative measures for voluntary uptake by the dog, such as hiding the tablet(s) in food or treats. If these methods failed then the owner could force feed the treatment or contact the investigator. The palatability of spinosad tablets was not assessed.

Owners were instructed to monitor dogs for one hour after dosing and to be alert for any evidence of vomiting, coughing, gagging, retching, drooling, salivating, or other adverse signs. The owner was instructed to contact the dispensing clinic to receive a replacement dose for any dog which vomited or regurgitated the tablet within 1 hour after dosing.

### Randomization and treatments

At each study site, qualifying households were assigned to a treatment group according to a randomized complete block design, with time of entry into the study as the blocking factor. Within blocks, dogs were randomly assigned in a 3:1 ratio to either the fluralaner group or the control group consisting of spinosad + amitraz. Because evidence of flea infestation was an enrollment criterion and because there are numerous approved flea control products, we decided against using a negative control group. Commercially available spinosad was chosen as a positive control product because at the time of study start this was the only FDA-approved oral product that provided flea knockdown and a month of anti-flea activity. This product does not have labeled activity against ticks, nor was there a FDA-registered product effective against a range of tick genera. As tick control would be required in some enrolling areas, we provided an amitraz collar due to its known effectiveness against ticks and absence of effect on fleas.

Each site had its own unique randomization table for assignment of households to treatment group; all dogs within a household received the same treatment. The randomization tables also included a scheme for random selection of a primary dog for households that had more than 1 dog with ≥ 10 live fleas at enrollment. The primary dog was managed identically to the other dogs, with the exception that the primary dog was the only dog in the household for which flea counting was performed for efficacy assessments at Visits 2, 3, and 4.

Fluralaner was administered at the label dose (25 – 56 mg/kg). Owners were instructed to administer treatment at meal time, immediately before the dog was offered its food. Additional doses of fluralaner tablets were dispensed at Weeks 12 and 24 in accordance with each dog’s body weight at those visits. Dogs in the control group received a 9% amitraz tick collar plus spinosad tablets at the labeled dose range of 30 to 60 mg/kg body weight. Spinosad tablets were also dispensed at Weeks 4 and 8 in accordance with each dog’s body weight measured at Weeks 4 and 8. The first administration of the allocated treatment(s) to any dog in the household, which occurred on the day of or shortly after the first visit, was classified as study Day 0.

### Justification of sample size

Sample size calculation was based on significant reduction of flea count from baseline at each time point. Using data from prior controlled studies demonstrating the effectiveness of fluralaner against fleas, power calculations indicated that no more than 10 households would be sufficient to provide 80% power to demonstrate statistically significant (p < 0.05) reduction of flea counts from baseline levels. To ensure broad regional inclusion of households and dogs, the study targeted enrollment of 100 households to be allocated to the fluralaner treatment group and 33 households to the control (spinosad + amitraz) group. The 3:1 enrollment ratio provided sufficient households in the control group for reference and comparison while maximizing the opportunity for post-treatment safety observations in the fluralaner group.

### Efficacy assessments

All study dogs returned to the study site for assessment visits every 4 weeks from Days 0 to 84, with a window ± 2 days at the Week 4 visit and ± 3 days at subsequent visits. All sites were trained in study assessment procedures. Blinding of any staff involved in study assessments was maintained by having a non-blinded individual (either the dog’s owner or a non-blinded member of clinic staff) remove the tick collar from control dogs prior to performance of flea counts, FAD assessment, or physical examination. Non-blinded individuals were not permitted to perform study tasks (flea count, FAD assessment, physical examination) that required blinding. Whole body flea counts were performed using a flea comb for at least 15 minutes. If fleas were recovered during the last minute of the procedure, combing was continued for an additional 5 minutes until no fleas were recovered. All live fleas were counted. Personnel at sites were also trained to manually search for ticks using a whole body examination lasting for 5 minutes. During the study, any attached ticks collected during clinic visits or by owners between visits were placed into sealable alcohol-containing vials for later identification by an experienced tick study investigator. Collected tick data were observational, and none of the study products were assessed for effectiveness against ticks. Fleas and ticks were counted on primary dogs at all visits (Weeks 0, 4, 8, and 12). For non-primary dogs, the protocol limited these assessments to the enrollment visit.

The primary effectiveness criterion was based on live flea counts in primary dogs as the experimental unit at Weeks 4, 8, and 12 (Days 28, 56, and 84) compared to baseline counts. For assessment of other variables (safety/adverse events, FAD assessment, counting and identification of ticks, palatability of fluralaner flavored chewable tablets), each dog was the experimental unit.

The geometric mean live flea count of the primary dogs was calculated separately for fluralaner and control dogs for each time point (Days 28, 56, and 84). The percent reduction at each time point was calculated according to the equation:


where D_0_ = geometric mean at baseline, and D_x_ = geometric mean on Day x (x = 28, 56, or 84).

The geometric mean flea count data were transformed prior to analysis using the Y = log_e_(x + 1) transformation. The log-transformed data were analyzed by a mixed linear model with repeated measures including treatment, visit and treatment*visit as the fixed effects and site as the random effect with the primary dogs in household as the repeated subject.

The model least squares means were used for comparisons and were back-transformed to obtain the estimates of geometric mean flea counts. A Kenward-Rogers adjustment was used to determine the denominator degrees of freedom for hypothesis testing.

Comparisons were conducted within each treatment group between the pre-treatment count and Days 28, 56 and 84 as well as between treatment groups at Days 28, 56, and 84. A two-sided test was used for each comparison at significance level α = 0.05. Statistical analysis was performed with the software package SAS (SAS Institute Inc., Cary, NC, USA, release 9.3).

Treatment was considered effective at each time point if the mean live flea count reduction was 90% or greater compared to Day 0, and the mean counts at Days 28, 56, and 84 were statistically significantly different (P ≤ 0.05) from and lower than Day 0. The percentages of flea-free primary dogs (those with 0 fleas) at Days 28, 56, and 84 (Weeks 4, 8, and 12) were compared between fluralaner and spinosad + amitraz treatment groups using a two-sided test with significant level α = 0.05. A non-parametric asymptotic approach was used to obtain the estimates for the differences of the percentages between treatment groups. StatXact v9 was used to perform the summary and analysis. All enrolled and treated dogs were included in the analysis of clinical safety. Collected tick data were observational, and none of the study products were assessed for effectiveness against ticks.

Signs of FAD were evaluated for all study dogs at each clinic visit. Assessment was performed by a masked veterinarian, evaluating the severity (No Sign, Mild, Moderate, and Severe) and anatomic location of the following signs of FAD: erythema, alopecia, papules, scales, crusts, and excoriation. If a sign was present, the veterinarian also indicated if it was indicative of FAD. Finally, it was recorded if the dog had received any medications (e.g., steroids, antihistamines, etc.) that could affect the assessment of FAD. The presence of signs of FAD in dogs that had not received any interfering medications was summarized for both treatment groups. For each sign of FAD, the percentage of dogs with the sign (Mild, Moderate, or Severe) at enrollment that resolved (became No Sign) at Week 12 without any interfering medications was summarized.

### Safety assessments

All study dogs were monitored until at least Week 12 (Day 84) for safety assessments, including clinic visits every 4 weeks. Fluralaner-treated dogs were monitored through Week 26, including clinic visits every 4 weeks from Week 12 through Week 24 and a final study visit on Week 26, 2 weeks after final fluralaner dose. Owners were instructed to monitor closely each dog after treatment administration, and to watch for possible adverse events that might occur at any time during the course of the study. Owners were asked to document all unfavorable or unintended health events or any other observations, including the finding of attached ticks, in a study diary.

Blood and urine were collected from all control dogs at baseline (Day 0) and Week 12 (Day 84). Blood and urine were collected from all fluralaner-treated dogs at baseline (Day 0), Week 12, and Week 26. Samples were submitted for clinical pathology assessments (CBC, chemistry screen, including liver and kidney panels, and urinalysis) for determination of any potential adverse treatment effects.

## Results

Two hundred and ninety-four (294) dogs from 157 households were enrolled between August 2011 and December 2011 at 18 study sites, with 118 primary dogs assigned to receive fluralaner (plus an additional 106 household dogs) and 39 primary dogs assigned to receive spinosad + amitraz (plus an additional 31 household dogs). Dogs remained under study until June 2012. Approximately 50% (56/118 fluralaner and 19/39 control) of enrolled households in each group contained a single dog. Age, gender and weight of the two treatment groups were balanced (Table 1). Approximately one third of dogs in each group were described as mixed breed, with the most common breed identifications being Chihuahua (6.7% [15/224] of fluralaner group dogs; 5.7% [4/70] of spinosad + amitraz dogs), Jack Russell (4.0% [9/224] and 4.3% [3/70]), Labrador retriever (4.0% [9/224] and 2.9% [2/70]) and Boxer (3.1% [7/224] and 4.3% [3/70]).

**Table 1:** **Demographics of enrolled dogs and distribution of numbers of dogs in each household**

		Fluralaner	Spinosad + Amitraz
		n = 224	n = 70
Age (years)	Mean (SD)	5.1 (3.7)	5.3 (3.7)
	Range	0.2 - 15.0	0.3 - 13.6
Weight (lb)	Mean (SD)	37.3 (29.16)	38.7 (26.25)
	Range	4.4 - 152.0	4.6 - 83.6
Gender	Female, Intact	33 (14.7%)	14 (20.0%)
	Female Spayed	82 (36.6%)	24 (34.3%)
	Male, Intact	53 (23.7%)	9 (12.9%)
	Male Neutered	56 (25.0%)	23 (32.9%)
Distribution of Household Sizes (number of dogs)			
	1	56/118 (47.5%)	19/39 (48.7%)
	2	32/118 (27.1%)	12/39 (30.8%)
	3	21/118 (17.8%)	6/39 (15.4%)
	4	4/118 (3.4%)	1/39 (2.6%)
	5	5/118 (4.2%)	1/39 (2.6%)

In the population of household dogs, 8.0% (18/224) in the fluralaner group (including eleven primary dogs) and 11.4% (8/70) in the spinosad + amitraz group (including three primary dogs) were not included in the outcomes assessments completed on Day 84. One non-primary dog in the fluralaner group was removed at the owner’s request because it vomited following the first treatment; the other two dogs in the household remained in the study. All other removals were considered unrelated to treatment and were due to diverse reasons including dispensing error, unblinding, protocol violations including failure to return for scheduled visits, and two deaths – one dog was hit by a car and died, and another dog was euthanized after a pre-existing cardiac failure condition worsened.

Pre-treatment flea counts were similar between assigned treatment groups (Table 2). In both treatment groups, flea counts of the primary dogs were significantly reduced (P < 0.0001 and the ≥ 90% efficacy was exceeded) compared to baseline at each timepoint (Table 2). The percentage of primary dogs free of fleas in the fluralaner group was significantly different from that in the spinosad + amitraz group on Days 28 and 84 (P values <0.0001 and =0.0370, respectively); but was not significantly different on Day 56 (P value =0.1364).

**Table 2:** **Primary dog geometric mean flea counts, percentage reduction from baseline, and flea-free dogs for treated dogs at each visit**

	V1	V2	V3	V4
	(enrollment)	(Day 28)	(Day 56)	(Day 84)
**Number of Primary Dogs**				
Fluralaner	117	113	108	106
Spinosad + Amitraz	39	38	34	32
**Geometric mean flea count (95% CI)**				
Fluralaner	32.2 (27.1 – 38.2)	0.1 (0.0 – 0.2)	0.1 (0.0 – 0.1)	0.1 (0.0 – 0.1)
Spinosad + Amitraz	32.6 (23.6 – 44.9)	1.3 (0.7 – 2.0)	0.2 (0.0 – 0.4)	0.1 (0.0 – 0.2)
P-value for comparison*	0.9469	<0.0001	0.1766	0.2189
**% effectiveness (reduction from baseline)**				
Fluralaner	NA	99.7%	99.8%	99.8%
Spinosad + Amitraz	NA	96.1%	99.5%	99.6%
**% flea-free primary dogs**				
Fluralaner	NA	91.1%	95.4%	95.3%
Spinosad + Amitraz	NA	44.7%	88.2%	84.4%
P-value for comparison**	NA	<0.0001	0.1364	0.0370

“NA” indicates that the value or calculation is “not applicable”. No effectiveness comparison was performed at V1 and no primary dogs were flea-free at V1.

*- P-value for comparison of model least squares means parameter estimates.

**- P-value for comparison of percentages using non-parametric asymptotic approach.

No major treatment-related adverse events were reported in either the fluralaner or the spinosad + amitraz group. The most commonly reported adverse event in each group was represented by emesis which was reported in a greater percentage of dogs in the spinosad + amitraz group than in the fluralaner group (Table 3). There were no clinically relevant changes in complete blood count, blood chemistry or urinalysis variables in either treatment group.

**Table 3:** **Frequency of adverse events reported in dogs randomized to receive either fluralaner flavored chewable tablets or spinosad together with amitraz**

Adverse Event	Fluralaner, dogs observed over 26 weeks	Spinosad + Amitraz, dogs observed over 12 weeks
	(N = 224)	(N = 70)
Emesis	16 (7.1%)	10 (14.3%)
Decreased appetite	15 (6.7%)	0 (0.0%)
Diarrhea	11 (4.9%)	2 (2.9%)
Lethargy	12 (5.4%)	5 (7.1%)
Polydipsia	4 (1.8%)	3 (4.3%)
Flatulence	3 (1.3%)	0 (0.0%)

At baseline (enrollment visit), in sites in Florida, Missouri, Alabama, Pennsylvania and Louisiana, a total of 47 attached ticks were found on 13 dogs assigned to the fluralaner group and 11 attached ticks were found on three dogs assigned to the spinosad + amitraz group. At Visit 2, one fluralaner dog had one dead non-engorged tick removed and at Visit 3 another fluralaner dog had two ticks (one alive non-engorged and one dead non-engorged tick) removed. At Visit 4, one control dog had one alive non-engorged tick removed.

In addition to the 62 attached ticks collected by investigators, 14 ticks were collected by owners and returned to the investigators. The 76 total ticks were submitted for tick identification. Across these sites, the identified tick genera were *Ixodes* (82.9% [63/76] of all submitted ticks), *Amblyomma* (13.2% [10/76]), *Dermacentor* (2.6% [2/76]) and *Rhipicephalus* (1.3% [1/76]).

Owner assessments of palatability of the fluralaner flavored chewable tablets were available for 559 of 621 doses administered over the course of the study. The palatability was consistent throughout the three doses (Table 4). Approximately 80% (451/559) of fluralaner doses were accepted free choice within 5 minutes after being offered and an additional 12.5% (70/559) were consumed with food or other treat. Therefore, a total of 92.5% of the fluralaner doses were consumed voluntarily by dogs in this study.

**Table 4:** **Palatability of three sequential treatments with fluralaner flavored chewable tablets administered at 12 week intervals**

Step when dose accepted by dog	Percentage of doses taken			
	1 ^st^Dose	2 ^nd^Dose	3 ^rd^Dose	Total
	~D0	~D84	~D168	
	(N = 207)	(N = 188)	(N = 164)	(N = 559)
**Free choice within 1 minute**	158 (76.3%)	142 (75.5%)	116 (70.7%)	**416 (74.4%)**
**Free choice within 1 – 5 minutes**	11 (5.3%)	15 (8.0%)	9 (5.5%)	**35 (6.3%)**
**Hidden in food or other treat**	27 (13.0%)	17 (9.0%)	26 (15.9%)	**70 (12.5%)**
**Dose force fed to dog**	10 (4.8%)	10 (5.3%)	13 (7.9%)	**33 (5.9%)**
**Investigator contacted for dosing**	1 (0.5%)	4 (2.1%)	0 (0.0%)	**5 (0.9%)**

Resolution of signs of FAD between enrollment and Week 12 (Day 84) in dogs not receiving medications that could affect the evaluation of FAD is summarized in Table 5. Relative to baseline, at Week 12 (Day 84) there was a high level of resolution of all signs of FAD in both groups.

**Table 5:** **Percentage of dogs with baseline flea allergy dermatitis signs that resolved without interfering medications at the final visit**

Flea allergy dermatitis sign	Fluralaner	Spinosad + Amitraz
Erythema	89.3% (50/56 dogs)	70.6% (12/17 dogs)
Alopecia	88.2% (30/34)	80.0% (8/10)
Papules	92.3% (12/13)	100.0% (7/7)
Scales	80.0% (16/20)	88.9% (8/9)
Crusts	95.5% (21/22)	100.0% (7/7)
Excoriation	91.7% (11/12)	100.0% (6/6)

## Discussion

A single fluralaner administration not only provided flea count reductions at least equivalent to three monthly spinosad treatments, but also resulted in significantly more dogs being flea-free at Week 4 (fluralaner 91.1% [103/113] vs spinosad 44.7% [17/38]) and at the final assessment at Week 12 (fluralaner 95.3% [101/106] vs spinosad 84.4% [27/32]) after the study began. This finding of high fluralaner efficacy parallels that reported from a European study in which the flea control, including the percentage of flea-free fluralaner-dogs at Week 4 post treatment, was superior to that provided by topical fipronil/(s)-methoprene [5]. Such rapid achievement of flea-free status in treated dogs, a status sustained for at least 12 weeks, points to fluralaner as providing a significant advance in the treatment and control of canine flea infestations.

These reductions in flea counts and high rate of complete flea elimination from study dogs translated into direct clinical benefits in alleviating signs of FAD. In each of the recorded signs of FAD, fluralaner treated dogs that enrolled with signs and had no interfering medications showed improvements of 80% (scales [16/20]) to 95% (crusts [21/22]). During protocol development, it was decided that this registration study would limit FAD assessments to those that could be objectively identified by investigators. Subjective assessments of owner-reported pruritus were therefore not completed. Nonetheless, because pruritus and other signs of FAD are caused by flea bites, and because both treatments were effective in eliminating fleas, it can be expected that associated benefits would include reductions in pruritus.

This was a real-world study designed to investigate the potential of owner-administered fluralaner tablets as a novel approach for controlling canine flea infestations. The study was open-label for owners and for clinic staff dispensing products, but all staff undertaking assessments remained blinded throughout. The only treatment instructions provided to owners were to administer the tablets before a meal and according to package directions with the one between-treatment protocol difference related to recording palatability of the fluralaner chewables. Two similar spinosad studies, only one of which assessed palatability (in a manner similar to our protocol) produced remarkably similar efficacy outcomes [3, 12]. The results from those studies are consistent with the results we report, indicating that palatability assessments did not affect results in any way.

At the enrollment visit, ticks were found on 24 study dogs, indicating that there would be some challenge with tick exposure during the study. Subsequent to treatment, few ticks were found, consistent with the reported effectiveness of the amitraz collar and supporting the sustained anti-tick activity of fluralaner that has been demonstrated in other field and laboratory studies [5, 7, 13].

The absence of serious adverse events (Table 3) indicates the safety of both products, with vomiting the most commonly reported adverse event (reported in 7.1% [16/224] of dogs treated three times with fluralaner tablets over the 26 week observation period (3 doses) and in 14.3% [10/70] of the control group dogs over the 12 week observation period (3 doses of spinosad). In other field studies of canine antiparasitic products, vomiting rates have varied substantially – rates as high as 18% and 15% have been reported to follow oral treatment of dogs with ivermectin tablets and ivermectin chewables, respectively, and a vomiting rate of 12% was reported to follow a single topical dose of selamectin or oral dose of spinosad [12, 14, 15]. Other adverse events were typically transient and mild, supporting the safety profile of spinosad and amitraz and indicating that fluralaner can be used safely in dogs. The detailed owner observations, the inconsequential adverse events associated with three sequential fluralaner treatments over 26 weeks, and uneventful laboratory analyses also confirmed earlier reports demonstrating the safety of fluralaner in dogs [5, 7–9].

## Conclusion

In conclusion, the results of this clinical study demonstrate that a single treatment of dogs with the fluralaner flavored chewable tablet, administered by owners, provides a level of flea control that is at least equivalent to that provided by 3 sequential treatments with spinosad tablets together with an amitraz collar. Linked to the high level of flea control, a substantial alleviation of associated clinical signs of FAD was also seen. The fluralaner flavored chewable tablets were found to be highly palatable for dogs. This study also demonstrated a favorable safety profile for fluralaner. The study therefore demonstrates that fluralaner flavored chewable tablets offer a significant advance in the treatment and control of canine flea infestations, providing sustained 12-week effectiveness with a single treatment.

## Abbreviations

FAD: Flea allergy dermatitis.
